# Research on Abuse in Home Care: A Scoping Review

**DOI:** 10.1177/15248380231165922

**Published:** 2023-04-20

**Authors:** Kevin Balkaran, Janice Linton, Malcolm Doupe, Kerstin Roger, Christine Kelly

**Affiliations:** 1 University of Manitoba, Winnipeg, MB, Canada

**Keywords:** workplace violence, community violence, elder abuse, mental health and violence

## Abstract

Home care is the preferred care option for most people who need support; yet abuse exists in these settings toward both home care workers and clients. There are no existing reviews that assess the scope of current research on abuse in home care, and tangentially related reviews are dated. For these reasons, a scoping review is warranted to map the current state of research on abuse in home care and examine current interventions in this field. Databases selected for searching were Medline and EMBASE on OVID, Scopus, and the following databases in EBSCOhost: Academic Search Complete, AgeLine, and Cumulative Index to Nursing and Allied Health Literature. Records were included if: (a) they were written in English; (b) the participants were home care workers or clients age 18 years or older; (c) they were published in journals; (d) they undertook empirical research; and (e) they were published within the last 10-year period. Following Graham et al. (2006), the 52 included articles are categorized as either knowledge inquiry or as intervention studies. We find three themes among knowledge inquiry studies: (1) prevalence and types of abuse in home care, (2) abuse in the context of living with dementia, and (3) working conditions and abuse. Analysis from the intervention studies suggest that not all organizations have specific policies and practices to prevent abuse, and no existing interventions to protect the well-being of clients were identified. Findings from this review can inform up-to-date practice and policymaking to improve the health and well-being of home care clients and workers.

Home care is the preferred care option for many as it enables people in need of care to live independently for as long as possible (Government of Canada, 2016). Yet, studies find that abuse exists in home care settings. Abuse is defined as a single or repeated act in a trusting relationship that causes distress among the recipient (World Health Organization, 2021). Abuse can include physical (violence), sexual, psychological, exploitation, aggressive, and neglectful acts (World Health Organization, 2021).

Existing studies suggest that home care workers and clients experience abuse. Home care workers refers to anyone who is paid to provide care for patients in their home, while home care clients refers to anyone who utilizes home care services. Sharipova et al. (2008) conduct a survey among home care workers in Danish municipalities and find acts such as hitting, scratching, being held, and being kicked by home care clients to be commonly reported. In another example, Geiger-Brown et al. (2007) study the effects of home care workers who experience abuse by clients in the United States and find that these workers are at an increased risk of developing depression. There is also existing research on client safety and maltreatment. Matthias and Benjamin (2003) study abuse toward clients in the United States and find home care workers neglect, injure, and steal from clients. Although the findings provide some insight into abuse in home care settings, these studies are dated, having taken place over 10 years ago.

Existing research suggests that the job satisfaction of home care workers is negatively impacted when they experience on-the-job violence. Canton et al.’s (2009) study on job satisfaction of home care workers in the United States finds that 26% of the workers that report experiencing violence disclose that they have low job satisfaction and consider leaving their work in home care altogether. In another example, Sherman et al. (2008) aim to understand the impact of hazards in the household on job satisfaction in the United States’ home care settings and find a positive correlation between job retention and job-related risks, such as verbal and physical abuse and the potential for violence.

Scholarly literature reviews on this topic exist, yet they employ a systematic approach. Nyberg et al. (2021) study the prevalence of physical, psychological, and gender-based violence among home care workers. Phoo and Reid (2022) examine recent evidence on violence toward home care workers. Clari et al. (2020) study sexual abuse toward home care workers by clients in home care settings. Hignett et al. (2016) explore risk factors for physical abuse toward both home care workers and clients. Schnelli et al. (2020) examine research on abusive incidents toward home care workers by clients living with dementia.

Systematic reviews examine a depth of existing knowledge on a particular issue (Munn et al., 2018). This design differs from a scoping review, which is used to map a range of existing evidence on a particular topic (McGowan et al., 2020; Munn et al., 2018). This study adds to the literature by employing a scoping review design to map the current state of research on abuse in home care.

Our approach is guided by concepts developed in the field of knowledge translation. Knowledge translation is described as the iterative process of advancing knowledge to improve health service delivery and the health and well-being of community members (Straus et al., 2009). Graham et al. (2006) outline a useful distinction between knowledge creation and action as part of the *knowledge to action* cycle. Knowledge creation includes knowledge inquiry (e.g., primary empirical studies), synthesis, and tools. Action refers to planned interventions that measure the effectiveness of different ideas. Knowledge translation allows researchers to identify gaps between knowledge and action, promoting changes to existing practices and policies that can enhance healthy behaviors (Straus et al., 2013). In light of these distinctions, the following questions guide this review: (a) what is known about abuse experienced by both home care workers and home care clients from recent empirical research studies? (b) what are the current intervention strategies to prevent or reduce abuse in paid home care settings? and (c) are they effective?

## Methods

This analysis is guided by a scoping review design. This scoping review is carried out through consulting the Preferred Reporting Items for Systematic Reviews and Meta-Analysis extension for Scoping Reviews (PRISMA-ScR) checklist, and incorporates the most recent methodological updates from the Joanna Briggs Institute (Peters et al., 2020; Tricco et al., 2018).

### Protocol

The scoping review team includes five members, with a three-person core team (Authors 1, 2, and 5) and two consulting members (3 and 4). Authors 1 and 5 work as independent screeners and author 2 is an experienced academic health sciences librarian with expertise in knowledge synthesis. Acting on the recommendation and advice from the two consulting members and author 5, author 1 drafted a protocol in November 2021 using the Preferred Reporting Items for Systematic Reviews and Meta-analysis Protocols checklist (Moher et al., 2015). This document was shared internally with Authors 2 and 5 to develop the searching strategy and eligibility criteria, respectively. The protocol is not registered.

### Identifying Relevant Studies

The search strategy was designed and carried out by the second author. Test searches identified keywords, subject headings, and the scope of the literature on this topic. Initially, test searches were carried out to focus on Canadian studies, but the number of results was too low and the team expanded to omit the geographic limit. This modification was consistent with the iterative process of scoping reviews (Levac et al., 2010). The databases selected for searching were Medline and EMBASE on OVID, Scopus, and the following databases in EBSCOhost: Academic Search Complete, AgeLine, and Cumulative Index to Nursing and Allied Health Literature. The InformIT network was not consulted during this review due to constraints in access. Records were included if: (1) they were written in English; (2) the participants were paid home care workers or adult home care clients (age 18 or older); (3) they were published in journals; (4) they conducted empirical (qualitative, quantitative, and/or mixed methods) research; and (5) they were published within the last 10-year period (from January 2011 to December 2021). Children (under 18) who experience abuse in home care were omitted from this review as they require a different framework of analysis. The timeframe was chosen to assess the current state of research in this field. Database searches were carried out by the first author in December 2021. Additional articles were identified by the first author through forward and backward citation linking from key articles retrieved through the database searches. Table 1 details the complete search strategy.

**Table 1.** Search Strategy.

Database	Search String	Number of Records
Scopus	(((TITLE(“home care” OR homecare OR “home nurs*” OR “home community care”) AND TITLE(violen* OR assault* OR aggress* OR crime* OR criminal* OR mistreat* OR abus*))) OR ((TITLE(“home care” OR homecare OR “home nurs*” OR “home community care”) AND ABS(violen* OR assault* OR aggress* OR crime* OR criminal* OR mistreat* OR abus*)))) AND NOT (TITLE(“out of home”)) AND ( LIMIT-TO ( DOCTYPE,“ar” ) OR LIMIT-TO ( DOCTYPE,“re” ) ) AND ( LIMIT-TO ( LANGUAGE,“English” ) ) AND ( LIMIT-TO ( PUBYEAR,2021) OR LIMIT-TO ( PUBYEAR,2020) OR LIMIT-TO ( PUBYEAR,2019) OR LIMIT-TO ( PUBYEAR,2018) OR LIMIT-TO ( PUBYEAR,2017) OR LIMIT-TO ( PUBYEAR,2016) OR LIMIT-TO ( PUBYEAR,2015) OR LIMIT-TO ( PUBYEAR,2014) OR LIMIT-TO ( PUBYEAR,2013) OR LIMIT-TO ( PUBYEAR,2012) )	52
EBSCOhost	TI ( “home care” OR homecare OR “home nurs*” OR “home community care” ) AND AB ( violen* OR assault* OR aggress* OR crime* OR criminal* OR mistreat* OR abus* ) OR TI ( “home care” OR homecare OR “home nurs*” OR “home community care” ) AND SU ( violen* OR assault* OR aggress* OR crime* OR criminal* OR mistreat* OR abus* ) NOT TI (“out of home”) OR TI (out n2 home).	46
Ovid Medline	1 exp Home Care Services/ 2 exp Violence/ 3 1 and 2 4 (comment or editorial or news or newspaper article).pt. 5 (letter not (letter and randomized controlled trial)).pt. 6 4 or 5 7 3 not 6 8 out of home.ti. 9 7 not 8 10 limit 9 to english language 11 limit 10 to yr=“2011 - 2021” 69	69
Ovid Embase	1 exp home care/ 2 exp violence/ 3 1 and 2 4 out of home.ti. 5 3 not 4 6 limit 5 to (article or article in press or “review”) 7 limit 6 to english language 8 limit 7 to yr=“2011 - 2021”	280

*Note*. Limitations: Published from 2011 to current. Limit to articles.

### Evidence Screening and Selection

There were two stages to the screening process: (1) abstract and title screening and (2) full-text screening. The first and fifth authors executed the first stage of the screening phase. The screeners reviewed the titles and abstracts and excluded records that did not meet the eligibility criteria. Titles and/or abstracts that were unclear to the purpose of the study were forwarded to full-text review for further analysis. Consensus and discrepancies were addressed through collaborative discussions. In the second stage of screening, the first author led the full-text screening component of the review. Author 1 was responsible for the full-text screening of studies as both screeners were in such high agreement with the title/abstract screening process; both screeners were in concordance with the eligibility criteria.

### Data Extraction

Peters et al. (2020) outline instructions for extracting data when conducting a scoping review. The first author followed this guide to develop a data extraction form to ensure that only key information that aligned with this scoping review’s questions was extracted from the studies. The data extraction form was routinely updated in an iterative process throughout this phase of data analysis. The key pieces of information extracted from the included studies were: paper, objective, methodology (qualitative, quantitative, and/or mixed methods), sample size, country of setting, data collection instruments, research design, and critical findings. Microsoft Excel software and Zotero reference manager facilitated record keeping.

### Synthesis of Results

The extracted data was synthesized according to the review questions. Open and axial coding techniques supported the extraction of data from the included studies, which were examined through thematic analysis, a form of data analysis used to investigate and examine patterns that emerge from the research (Auerbach, 2003; Seers, 2012). Thematic analysis is credited as a useful approach in scoping reviews as it identifies key characteristics related to a concept (Alessi et al., 2021; Davy et al., 2016). In this scoping review, codes were developed during the full-text screening phase of the review. Codes were applied to the content from the included studies, where patterns were observed. Microsoft Word software stored excerpts from the studies and arranged them in accordance with themes.

## Results

The search strategy retrieved 460 records—447 from database searching and 13 from forwards and backwards searching. These records were converted into individual research information systems files and exported into Mendeley reference manager. Mendeley supports de-duplication, identifying and removing 100 duplicate records. After de-duplication, the titles and abstracts of the 360 identified studies were screened against the inclusion criteria, and 260 studies were excluded from consideration. The rate of agreement between the two screeners in the first stage was 90%.

**Figure 1.**
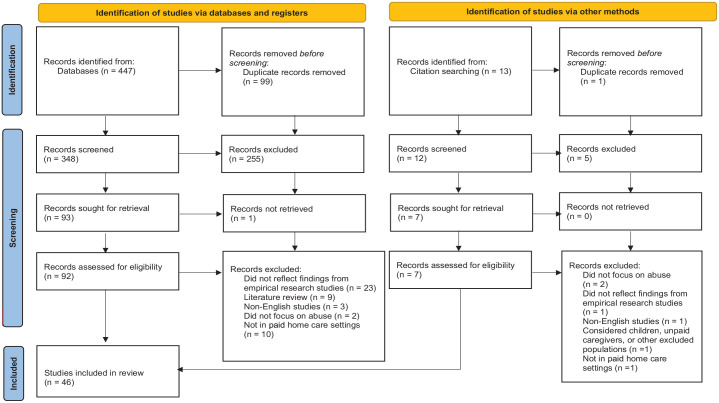
Preferred Reporting Items for Systematic Reviews and Meta-Analysis flow chart.

The remaining studies (*n* = 100) were forwarded to the full-text review stage of the screening process. At this stage, Author 1 read all 100 articles in-depth. A careful analysis of the content from these studies against the inclusion criteria led to the removal of 54 studies. These studies were excluded because they: did not reflect findings from empirical research studies; were literature reviews; were not written in English; did not focus on abuse; were not in paid home care settings (e.g., studies set in residential care or hospitals), and considered children, unpaid caregivers, or other excluded populations. One study was excluded as it was irretrievable from the University of Manitoba’s document delivery service. A total of 46 articles were included in this scoping review. The full study flow is presented in Figure 1 using the PRISMA-ScR diagram.

### Characteristics of Included Studies

**Figure 2.**
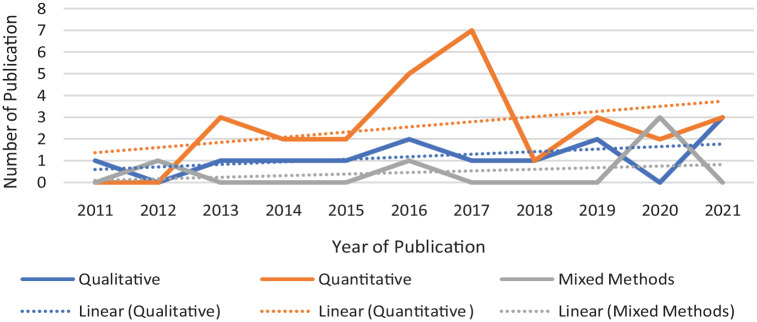
Distribution of studies by research methods.

The frequency of published studies on abuse in home care increased from 1 study in 2011 to 6 studies in 2021. This increase was found among qualitative and quantitative studies. Figure 2 outlines the frequency of included studies between 2011 and 2021, characterized by research approach. Overall, 28 studies identify as quantitative, 13 as qualitative, and 5 as mixed-methods studies.

Most (*n* = 21) of the included studies explore findings from empirical research set in the United States. Other countries included in this review are: Australia (*n* = 1), Canada (*n* = 4), Finland (*n* = 1), Germany (*n* = 2), India (*n* = 1), Israel (*n* = 5), Japan (*n* = 2), New Zealand (*n* = 1), Norway (*n* = 1), Rio De Janeiro (*n* = 1), South Korea (*n* = 2), and Switzerland (*n* = 4).

As described earlier, we broadly categorize the studies in accordance with Graham et al.’s (2006) distinction between knowledge creation and action. The majority (*n* = 31) of the 46 included studies are considered knowledge inquiry in this field. These studies produce insight into abuse toward home care workers and clients. Commonly applied data collection instruments include surveys, interviews (semi-structured and focus groups), and secondary review of non-academic documents (e.g., reports of abuse). Three study themes are identified among current knowledge inquiry research; there are studies on: (1) prevalence and types of abuse in home care, (2) abuse in the context of living with dementia, and (3) working conditions and abuse.

The remaining (*n* = 15) studies contribute to our body of knowledge on interventions to protect home care workers. These studies assess the effectiveness of abuse-preventing interventions in home care by experimental designs, and make observations on the current state of interventions, briefly touching upon a lack of interventions to protect home care clients. Surveys and interviews are commonly cited data collection approaches in this set. A complete summary of research studies is found in Supplemental Appendix A. Supplemental Appendix B summarizes the critical findings of each included study.

### Knowledge Inquiry

#### Prevalence and types of abuse in home care

Findings from the literature suggest that there are several types of abuse which occur in the context of home care settings. These types of abuse include verbal, sexual, physical, and acts of neglect. This theme can further be broken down to illustrate how these forms of abuse are unique toward home care workers and clients.

##### Abuse experienced by home care workers

Findings from the literature suggest that abuse toward home care workers is prevalent. Results from a questionnaire administered to home care workers in Israel finds that many home care workers experience some form of abuse while providing routine care services (Green & Ayalon, 2017). Further, there are certain risk factors that increase the likelihood that home care workers may experience abuse. Home care workers who belong to a younger age group, occupy temporary positions, have limited experience working in home care, and have limited time interacting with patients are more likely to experience abuse from clients in comparison to their counterparts (Byon et al., 2017c). Verbal abuse is the most commonly cited type of abuse toward home care workers (Choi et al., 2016; Fujimoto et al., 2019; Hanson et al., 2015; Ifediora, 2018; Quinn et al., 2016; Schnelli et al., 2021a, 2021b). To illustrate, Hanson et al. (2015) conduct a survey on home care workers in the United States and find that cases of verbal abuse comprised 51.5% of the total proportion of abuse reported in 2014. This form of abuse includes cursing or screaming at home care workers, questioning a home care worker’s competency or qualifications, and/or making insulting and unfavorable remarks, including but not limited to references to race (Schnelli et al., 2021a, 201b). Threats are also identified as a form of verbal abuse, as home care workers frequently cite being threatened by clients in their homes (Byon et al., 2016; Schnelli et al., 2021b).

Other less commonly reported forms of abuse toward home care workers include sexual harassment and physical abuse (Byon et al., 2017b; Hanson et al., 2015; Schnelli et al., 2021a). Schilgen et al. (2019) find 14% of the German sample report experiencing sexual harassment. Nakaishi et al. (2013) conducted focus groups and interviews in the United States and find that some organizations have little, if any support for home care workers who experience sexual harassment, with many home care workers forced to choose between reporting their experiences to the police, or resign from their positions. The findings of physical abuse toward home care workers are limited; in a quantitative study in the United States, Byon et al. (2016) find that 2.5% of home care workers report experiencing physical abuse from their clients. According to a qualitative study set in Rio De Janeiro, home care clients are more likely to become physically abusive toward home care workers when they experience infections, pain, or delirium (Capeletto et al., 2021).

##### Abuse experienced by home care clients

Although limited, findings from this review suggest abuse is prevalent toward home care clients, and in particular, older adults. Ayalon (2016) studied elder neglect in home care settings in Isreal. The findings suggest that older home care clients who have little interactions/estranged relationships with family members, and live alone are more likely to experience neglect.

#### Abuse in the context of living with dementia

The second theme is studies on abuse related to dementia caring. Caring for, and living with dementia influences the caring dynamic in the context of home care. Home care clients living with dementia, and home care workers who provide support, experience abuse differently from the general home care population, meriting its own independent analysis.

##### Abuse toward home care workers by clients with dementia

Home care workers and supporting clients living with dementia are more likely to experience abuse in comparison to their counterparts. This is due to the necessary increase in the amount of support for, and the unpredictable behavioral nature of people living with dementia (Byon et al., 2016; Green & Ayalon, 2017; Schnelli et al., 2021b). A quantitative study set in Israel finds home care workers who support people with dementia report work-related abuse more often, with as much as a 145% increase in experiencing abuse compared to workers who do not care for clients with dementia (Green & Ayalon, 2017). Abuse from home care clients living with dementia toward formal and informal caregivers threaten their well-being. A quantitative study set in Canada finds clients with dementia plus traumatic brain injury (TBI) were significantly more likely to be physically abusive toward distressed caregivers, compared to clients with dementia but without TBI (Vu et al., 2014).

Home care workers who support clients living with dementia employ behavioral techniques to protect themselves from abuse. Changing the physical environment is the most common technique as it alters social dynamics; for example, a qualitative study set in Canada finds home care workers tend to avoid intimate spaces when providing care for clients, such as bedrooms, as these locations are associated with increased agitation from people living with dementia (Herron & Wrathall, 2018). Further, home care workers remove clients with dementia from noisy and congested environments, and other environments with heavy movements as these environments trigger abusive actions from clients (Herron & Wrathall, 2018).

Some studies find clients living with dementia are often verbally abusive toward home care workers (Fujimoto et al., 2019; Schnelli et al., 2021b; Wang et al., 2016). Yet, the way in which clients living with dementia express verbal abuse is unique. Herron and Wrathall (2018) finds that people living with dementia vocalize their feelings and emotions to express themselves. Clients with dementia are just as likely to act physically abusive toward themselves as toward others; a mixed-methods study set in Switzerland finds clients are as likely to hit, kick, and scratch themselves as they attempt to carry out these acts against home care workers (Schnelli et al., 2021b).

##### Abuse toward home care clients with dementia by home care workers

There is evidence that suggests home care workers abuse clients living with dementia. Home care workers express verbal frustration by “hollering back” at clients with dementia (Herron & Wrathall, 2018). Friedman et al. (2015) conduct a quantitative study in the United States and find that clients living with dementia are dependent on their home care workers for support; for example, clients who are unable to make complex decisions rely on their home care workers for medical management. Home care workers recognize this dependency and use it to shift power dynamics between the group by utilizing manipulative and other abusive techniques.

#### Studies on working conditions and abuse

The third study theme examines the link between working conditions and abuse. This theme illustrates the similarities and differences among migrant and local home care workers who experience abuse. We also examine how communication breakdowns and stressful job demands contribute to a normalized culture of abuse in home care settings.

##### Abuse toward migrant home care workers

Migrant home care workers, that is, home care workers who move from one country to another to obtain work, are especially vulnerable to exploitation from their employers. Studies cite employment barriers that challenge the well-being of migrant home care workers. Migrant home care workers report they do not receive days off, sick days, or holiday vacations, a contract which outlines their working conditions and rights to financial compensation, and can be forced to work in isolation with limited supervision (Green & Ayalon, 2016, 2018; Schilgen et al., 2019). Live-in migrant home care workers who reside with their clients in Israel were surveyed, and the results suggest they are often unable to distinguish working hours, citing that they work “around the clock” (Green & Ayalon, 2018). Migrant home care workers are reluctant to report abuse as they tend to believe it is a private matter; reporting abuse takes too much time and effort; they are afraid the abuse will get worse if they report incidents, and/or they wished not to harm others through reporting (Ayalon, 2012; Green & Ayalon, 2016, 2018; Schilgen et al., 2019). Risk factors for experiencing abuse toward migrant home care workers include language and communication barriers, limited experience working in home care settings, and being asked to do more than the requirements of the jobs (Ayalon, 2012; Green & Ayalon, 2016, 2018). A quantitative study in Israel finds migrant home care workers with little financial independence to be more likely to stay with employers and endure exploitation and other forms of abuse rather than to resign (Green & Ayalon, 2017).

##### Abuse toward local home care workers

Similar findings are cited among home care workers employed in their country of origin. Employers can exploit local home care workers; for example, home care workers living in Israel cite not receiving vacation time or sick leave (Green & Ayalon, 2018). Home care workers in Germany consistently report experiencing stress and that they are unable to distance themselves from their work due to the physical and psychological demands of working in home care (Schilgen et al., 2019). Yet, other studies claim high rates of job satisfaction among home care workers employed in their own country; work autonomy supported by flexible schedules and the freedom to work independently, with employment security and opportunities for promotion and professional advancement contribute to higher job satisfaction from local home care workers living in the United States (Quinn et al., 2016).

##### A normalized culture of abuse

Several factors contribute toward shaping a workplace which normalizes the experience of abuse. Breakdown in communications can contribute to a culture of silence on-the-job despite experiencing abuse toward home care workers; home care workers who work alone feel disconnected and unsupported by colleagues and managers, are unaware that other home care workers also experience abuse, and are less likely to report abuse (Grasmo et al., 2021; Möckli et al., 2020; Nakaishi et al., 2013). Clients expect home care workers to complete any and all tasks assigned to them. Conflicts arise when home care workers push back on extra tasks, oftentimes escalating to verbal abuse by clients (Kim et al., 2013).

Stressful job demands are associated with poor health outcomes among home care workers. At any moment, clients may engage in violent outbursts toward home care workers, who must remain professional and not take these outbursts personally (Markkanen et al., 2014; Nakaishi et al., 2013). The psychological well-being of home care workers is compromised by clients who display abusive behavior toward them during routine care (Abey-Nesbit et al., 2021; Womack et al., 2020). Home care workers who fear experiencing abuse and have poor relationships with clients cite negative health impacts such as insomnia, burnout, and anxiety (Grasmo et al., 2021; Hanson et al., 2015; Ifediora, 2018; Möckli et al., 2020). A quantitative study set in the United States finds home care clients utilize sharp objects such as intravenous needles as weapons towards home care workers, and these home care workers experience both verbal and physical injury from agitated clients (Brouillette et al., 2017). Clients who consume excessive amounts of alcohol create difficult working conditions for home care workers; a qualitative study in Finland finds home care workers are forced to work through their own psychological trauma associated with a history of alcohol when solving problematic situations involving heavily intoxicated clients (Koivula et al., 2016).

### Action: Intervention Studies

The second major category of studies are intervention studies seeking to address abuse in home care settings, and is based on 15 studies. Some organizations offer structural supports to intervene in abusive situations. Study findings support the existence of written policies and practices to deter abuse in home care (Gross et al., 2013) and provide home care workers with training on workplace violence (Singh et al., 2020). A study set in the United States finds that new hires must complete abuse prevention training programs as part of their orientation, and maintain ongoing training on this topic to upwards of 1 year after working in home care (Vladutiu et al., 2016). A qualitative study in the United States finds that some organizations facilitate access to cell phones among home care workers to contact emergency authorities in situations of violence (Bien et al., 2021).

Experimental studies assess the effectiveness of interventions that aim to reduce and prevent abuse toward home care workers. Dyer and Abildso (2019) evaluated a training program on abuse in home care. The evaluation is aimed at assessing the knowledge, skills, and abilities of home care workers in relation to recognizing abuse prior to and after receiving the training. Findings from this evaluation suggest that the training program is responsible for a significant improvement of home care worker’s knowledge, skills, and abilities to respond to situations of violence in home care. Glass et al. (2017) examined the effectiveness of a computer-based abuse prevention training program for female home care workers in the United States and find female home care workers reporting an improved level of confidence to respond to verbal abuse as a result of participating in the training program. Kurata and Ojima (2014) assess family caregivers’ and home care providers’ knowledge of physical restraints used with elders living at home in Japan and find that home care workers are more knowledgeable in these techniques.

Observational studies of organizations that implement intervening strategies to reduce abuse report promising findings. Home care workers who engage in regular coaching sessions with management are able to reduce abusive interactions from clients by learning strategies to protect themselves such as ending visits early if interactions became violent (Byon et al., 2017a; Gross et al., 2013). A quantitative study in the United States finds that home visits accompanied by police or security escorts and having co-workers accompany the care worker during routine care visits can reduce the prevalence of abuse toward home care workers (Gross et al., 2013).

Yet, not all organizations employ intervention strategies to protect home care workers. In a quantitative study set in Germany, Rabold and Goergen (2013) cite that abuse toward home care workers will be reduced if organizations equip their staff with proper training on how to deal with abusive incidence. Kim et al. (2020) find that organizations lack reporting systems to protect home care workers from abuse by clients in South Korea. Further, organizations do not effectively address situations of abuse after the incident occurs. Schnelli et al. (2021c) conducted a quantitative study in Switzerland and find abuse management training is not available for some home care workers, and no reporting system exists to deal with abusive incidents.

The research also suggests that there is a lack of intervention strategies to protect home care client, yet these findings are limited. A qualitative study in Canada finds clients who are unable to screen visitors accidentally invite imposters who pose as home care workers into their home (Macdonald et al., 2011). A lack of screening interventions for clients may increase a client’s vulnerability to experience abuse.

Opportunities exist to ensure these organizations meet the safety demands of their home care workers and clients. Among these include regularly evaluating programs, incorporate training on self-neglect among older clients, establish reporting practices, and employ positive incentives to encourage the reporting of abuse (Byon et al., 2020; Gross et al., 2013; Johnson, 2015; Markkanen et al., 2017). Organizations should also take steps to reduce the stigma of reporting abuse by prioritizing more time during internal meetings to develop interventions aimed at reducing the prevalence of abuse (Couture et al., 2019).

## Discussion

The purpose of this scoping review is to map the current state of research on abuse in home care. Included studies are categorized according to the Graham et al. (2006) distinction between knowledge creation and action. Knowledge inquiry is a concept of knowledge creation, and three themes are identified; there are studies on: (1) prevalence and types of abuse in home care, (2) abuse in the context of living with dementia, and (3) working conditions and abuse. Action includes knowledge intervention studies that assess the effectiveness of, and make observations on the current state of interventions. Findings from this review provide a body of evidence to inform up-to-date practices and policy making in home care, and identify gaps for future research opportunities.

The first knowledge inquiry theme is prevalence and types of abuse in home care. This theme examines the frequency of abuse experienced toward and from home care workers, and the types of abuse that are experienced by these groups. For example, home care workers in particular experience physical, verbal, and sexual abuse, with some more commonly reported than others (Byon et al., 2017b; Choi et al., 2016; Fujimoto et al., 2019; Hanson et al., 2015; Ifediora, 2018; Karlsson et al., 2019; Quinn et al., 2016; Schnelli et al., 2021a, 2021b). This body of evidence illustrates that a significant amount of research has been conducted to determine how home care workers experience abuse. Interestingly, the authors found that there is a limited number of articles that addressed abuse toward home care clients, suggesting a dearth in research and an avenue for future analysis.

Abuse in the context of living with dementia is the second knowledge inquiry theme. This review finds that clients living with dementia and their home care workers encounter experiences of abuse that are unique in comparison to those where dementia is absent. We observed a higher prevalence of abuse among home care workers who provide care to clients living with dementia (Byon et al., 2016; Green & Ayalon, 2017; Schnelli et al., 2021b). We also find that home care workers employ unique methods to respond to abuse, clients living with dementia have a distinctive style of displaying abuse, and home care workers shift power dynamics to manipulate clients (Herron & Wrathall, 2018).

As a result of manipulation, home care clients living with dementia may change the way they act around home care workers. For example, clients living with dementia who experience manipulation may distrust or fear those in their environment, only keeping to themselves (Dexter, 2022). Over time, manipulation can deteriorate a client’s self-worth, leading to poor mental and physical health and well-being.

The third knowledge inquiry theme is working conditions and abuse. Migrant home care workers are particularly vulnerable to experiencing abuse either by clients or through a poor working environment created by the employer (Green & Ayalon, 2016, 2018; Schilgen et al., 2019), warranting an assessment of existing policies and guidelines to protect the well-being of this group. Yet, conflicting findings on abuse toward local home care workers, those working in their country of origin, on a global scale might suggest countries employ different practices to handle abuse (Green & Ayalon, 2018; Quinn et al., 2016).

Action is the second concept from Graham et al. (2006) and can be characterized as intervention studies. Experimental studies that aim to assess the performance of interventions, such as computer-based and other training programs, show promise toward protecting home care workers who experience abuse in home care settings (Glass et al., 2017). Observational studies report similar findings, with emphasis on coaching sessions between home care workers and management (Byon et al., 2017a; Gross et al., 2013) Yet, we learnt that not all organizations employ interventions to prevent abuse in home care, with some studies citing no guidelines to educate their home care workers on how to handle abuse (Byon et al., 2020; Gross et al., 2013; Johnson, 2015; Markkanen et al., 2017)

We are concerned to find only a limited number of studies that reference employer-based actions to protect the well-being of clients, with no concrete examples of existing policies. As such, more assessment of interventions to protect clients may be needed using evaluation designs. Assessing interventions can create specific and dependable knowledge on abuse toward clients through identifying cause and effect relationships (Palys & Atchison, 2013). This knowledge can inform practices to protect the health and well-being of clients.

## Limitations

Only studies published in English are included in the eligibility criteria for the review. The screeners did identify studies where the title and abstracts were written in English, but the body of the article was written in a different language, which disqualified these articles from consideration. These studies may have contributed to the synthesis of this review. Additionally, only the first author was tasked with assessing the full-text eligibility of articles to respect the project timeline due to capacity and workload constraints amongst the authors. This decision implies the existence of selection bias during this stage in the review, where the sole screener was in direct control of determining which studies were included for data extraction and analysis. However, both screeners felt that this decision was justified given the high level of agreement during title and abstract screening and that the mutually agreed upon eligibility criteria illustrated concordance between the two screeners. Finally, although the grey literature may identify interventions to protect the well-being of home care workers and clients, they were not included in this review as the scope was limited to peer-reviewed literature.

## Conclusion

This scoping review summarizes the current state of research and interventions related to abuse in home care. Key concepts and limitations are identified in the research, producing a body of evidence to inform up-to-date practice and policymaking. Table 2 summarizes the implications for practice, policy, and research.

**Table 2.** Summary of Implications for Practice, Policy, and Research.

Practice	• Facilitate volunteering programs between schools and home care where students can spend time with clients whose health is compromised due to the effects of neglect. • Introduce an “intergenerational system” where inexperienced care workers are accompanied by skilled care workers to encourage building rapport with client. • Employers/managers should set regularly occurring meetings that primarily focus on the current state of abuse in their organization. • All care workers should receive regular training on abuse prevention from their employers.
Policy	• Develop policies to address the experiences of migrant care workers (as outlined in this review). • Establish anonymous reporting guidelines for clients who fear about the consequences of reporting their care workers. • Create peer groups and other supportive environments for workers to come together and share their experiences of abuse in a safe environment.
Research	• Promote more research on abuse toward home care clients. • Promote qualitative studies of abuse in home care to incorporate more examples of lived experiences of abuse.

The authors identify three areas for future practice. Drawing upon the findings from Grasmo et al. (2021), an “intergenerational system” can be introduced, where inexperienced home care workers are accompanied by skilled home care workers during routine care visits to encourage rapport between clients. This practice can avoid aggression among clients who perceive new home care workers as inexperienced. It can serve as an ongoing mentorship role, as issues arise over time. Second, employers/managers can destigmatize the perception of abuse through setting regularly occurring meetings that focus on education, raising awareness, offering strategies, and promoting discussion about possible abuse in their organization. This suggestion is in response to the findings from Couture et al. (2019) which references the stigma surrounding abuse in home care settings. Finally, to address Rabold and Goergen’s (2013) theory that proper training will reduce abuse toward home care workers, regular workplace training should be made readily available for workers and made mandatory as an ongoing requirement for employment. Participating in training will ensure that all workers are equipped with the knowledge to prevent abuse, and allow them to stay current with the reality of abuse in home care.

In the context of home care, policies aim to protect the health and well-being of home care workers and clients. A lack of organizational support (Schnelli et al., 2021c) suggests the need to develop policies that address abuse and exploitation of migrant and local home care workers, who face different and overlapping issues in this context. Guidelines should be in place to prevent organizations from exploiting migrant home care workers (Green & Ayalon, 2016, 2018; Schilgen et al., 2019) such as the development of advocacy groups that collaborate with migrant workers and organizations to facilitate beneficial working conditions. Further, we advocate for anonymous reporting guidelines for clients who fear the consequences of reporting their home care workers (Ayalon, 2012; Green & Ayalon, 2016, 2018; Schilgen et al., 2019). This measure will ensure their needs are addressed while at the same time protecting their well-being. Finally, building on the findings from Green and Ayalon (2017) that suggest abused workers work in isolation, we argue for the development of peer groups and other supportive environments for workers to come together and share their experience of abuse in a safe environment. Supportive environments can foster resiliency and self-worth among home care workers, improving their well-being.

There are a limited number of studies that address abuse toward clients in comparison to home care workers; 13 percent of the total number of articles reviewed addressed abuse toward home care clients. Funding studies and interventions targeting abuse toward home care clients can contribute to the development of knowledge in this field and inform evidence-based action aimed at protecting their well-being. Further, more mixed methods research on abuse in home care should be encouraged. Mixed methods research can employ generalizable quantitative findings to complement qualitative data. The benefits of mixed-methods research, along with the lack of this design in recent studies of abuse in home care settings may encourage researchers in various countries to employ this methodology in subsequent research. Taken together, these opportunities for future research will make a rich contribution to knowledge on abuse in home care.

## Supplemental Material

sj-docx-1-tva-10.1177_15248380231165922 – Supplemental material for Research on Abuse in Home Care: A Scoping Review Supplemental material, sj-docx-1-tva-10.1177_15248380231165922 for Research on Abuse in Home Care: A Scoping Review by Kevin Balkaran, Janice Linton, Malcolm Doupe, Kerstin Roger and Christine Kelly in Trauma, Violence, & Abuse
