# Supplementary material for: Research on Abuse in Home Care: A Scoping Review
Source: Trauma Violence Abuse. 2023 Apr 20;25(2):885–97. doi: 10.1177/15248380231165922 (PMC10913332; doi:10.1177/15248380231165922)
Supplement: sj-docx-1-tva-10.1177_15248380231165922 – Supplemental material for Research on Abuse in Home Care: A Scoping Review [file sj-docx-1-tva-10.1177_15248380231165922.docx]

**Supplementary File**

| **Appendix A**  *Summary of Included Research Studies* | | | | | | | |
| --- | --- | --- | --- | --- | --- | --- | --- |
| **Knowledge Inquiry Studies** | | | | | | | |
| **Paper** | **Themes** | **Data Collection Instrument** | **Sample** | **Methodology** | **Date of Data Collection** | **Abuse Towards**  **(Workers/**  **Clients)** | **Research Question Addressed *** |
| Abey-Nesbit et al., 2021 | (3) Relationships among working conditions and abuse | Collection of existing cross sectional data | N = 105,978 peoples aged 65 and older who use home care | Quantitative | 2012-18 | Workers | (1) |
| Ayalon, 2012 | (3) Relationships among working conditions and abuse | Focus group interviews | N = 178 (Filipino home care workers) | Mixed Methods | Unknown | Workers | (1) |
| Ayalon, 2016 | (1) Prevalence and types of abuse in home care | Interviews | N = 1,597 (686 family members, 388 older adults, 523 home care workers) | Qualitative | Unknown | Clients | (1) |
| Brouillette et al., 2017 | (3) Relationships among working conditions and abuse | Questionnaire | N = 1,178 (home care workers) | Quantitative | 2012-13 | Workers | (1) |
| Byon et al., 2016 | (1) Prevalence and types of abuse in home care,  (2) Abuse in the context of living with dementia | Survey | N = 876 (home care workers) | Quantitative | 2006 | Workers | (1) |
| Byon et al., 2017a | (1) Prevalence and types of abuse in home care | Secondary data from national survey | N = 3,377 (home care workers) | Quantitative | Unknown | Workers | (1) |
| Byon et al., 2017b | (1) Prevalence and types of abuse in home care | Survey | N = 964 (home care workers) | Quantitative | 2006 | Workers | (1) |
| Capeletto et al., 2021 | (1) Prevalence and types of abuse in home care | Interviews, observations | N = 162 (older adults in a home care program) | Qualitative | 2018 | Workers | (1.2.3) |
| Choi et al., 2016 | (1) Prevalence and types of abuse in home care | Survey | N = 55 (home care workers) | Quantitative | Unknown | Workers | (1) |
| Friedman et al., 2015 | (1) Prevalence and types of abuse in home care,  (2) Abuse in the context of living with dementia | Questionnaires, Interviews | N = 724 (patients receiving care at home from nurse) | Quantitative | Unkown | Clients | (1) |
| Fujimoto et al., 2019 | (1) Prevalence and types of abuse in home care    (2) Abuse in the context of living with dementia | Questionnaires | N = 184 (home visit nurses) | Quantitative | 2012-13 | Workers | (1,2) |
| Grasmo, Liaset & Redzovic, 2021 | (3) Relationships among working conditions and abuse | Semi-structured interviews | N = 8 (home care workers) | Qualitative | 2020 | Workers | (1) |
| Green & Ayalon, 2016 | (3) Relationships among working conditions and abuse | Questionaire | N = 187 (Filipino home care workers) | Quantitative | Unknown | Workers | (1,2) |
| Green & Ayalon, 2018 | (3) Relationships among working conditions and abuse | Survey | N = 523 (338 migrant live-in home care workers and 185 local live-out home care workers) | Quantitative | 2014 | Workers | (1) |
| Green & Ayolon, 2017 | (1) Prevalence and types of abuse in home care  (2) Abuse in the context of living with dementia  (3) Relationships among working conditions and abuse | Questionnaire | N = 187 (Filipino home care workers) | Quantitative | Unknown | Workers | (1) |
| Hanson et al., 2015 | (1) Prevalence and types of abuse in home care  (3) Relationships among working conditions and abuse | Survey | N = 1,214 (female home care workers) | Quantitative | Unknown | Workers | (1) |
| Herron & Wrathall, 2018 | (2) Abuse in the context of living with dementia | Semi-structured interview | N = 27 (people who care for dementia patients) | Qualitative | 2016-17 | Workers | (1) |
| Ifediora, 2018 | (1) Prevalence and types of abuse in home care  (3) Relationships among working conditions and abuse | Survey | N = 300 (doctors who perform house visitations) | Quantitative | 2013-14 | Workers | (1) |
| Kim, Noh, & Muntaner, 2013 | (3) Relationships among working conditions and abuse | Survey | N = 1,599 (homecare workers) | Quantitative | 2003-04 | Workers | (1) |
| Koivula et al., 2016 | (3) Relationships among working conditions and abuse | Semi-structured interviews | N = 10 (home care workers) | Qualitative | 2014-15 | Workers | (1) |
| Kurata, & Ojima, 2014 | (1) Prevalence and types of abuse in home care | Survey | N = 944 (494 family care worker, 201 home helpers, 78 visiting nurses, 131 visiting physicians, and 158 care managers of home-dwelling frail elders needing some care and medical support) | Quantitative | 2007-09 | Workers | (1,2) |
| Markkanen et al., 2014 | (3) Relationships among working conditions and abuse | Focus group | N = 12 (focus group interviews with home care aides) and 26 (in depth interviews with 15 home care agencies and 11 home care clients) | Qualitative | 2011-12 | Workers | (1) |
| Möckli et al., 2020 | (3) Relationships among working conditions and abuse | Survey | N = 6 (home care agencies) | Quantitative | 2017-18 | Workers | (1) |
| Nakaishi et al., 2013 | (1) Prevalence and types of abuse in home care  (3) Relationships among working conditions and abuse | Focus groups and interviews | N = 193 (83 home care workers, 99 HR employees, 11 consumer employees) | Qualitative | 2009-10 | Workers | (1) |
| Quinn et al., 2016 | (1) Prevalence and types of abuse in home care  (3) Relationships among working conditions and abuse | Survey | N = 1249 (home care aides) | Quantitative | Unknown | Workers | (1) |
| Schilgen et al., 2019 | (1) Prevalence and types of abuse in home care  (3) Relationships among working conditions and abuse | Semi-structured interviews | N = 48 (24 migrant and 24 autochthonous nurses) | Qualitative | 2017 | Workers | (1) |
| Schnelli et al., 2021a | (1) Prevalence and types of abuse in home care  (2) Abuse in the context of living with dementia | Secondary review of existing documents | N = 1,186 (nursing documentation) | Mixed Methods | 2019 | Workers | (1) |
| Schnelli, et al., 2021b | (1) Prevalence and types of abuse in home care | Survey | N = 842 (healthcare professionals) | Quantitative | 2019 | Workers | (1) |
| Vu et al., 2014 | (2) Abuse in the context of living with dementia | Collection of existing cross sectional data | N = 488 290 (home care clients) | Quantitative | 2003-10 | Workers | (1) |
| Wang et al., 2016 | (2) Abuse in the context of living with dementia | Secondary analysis of records | N = 28,475 (records of home care patients) | Mixed Methods | 2010 | Workers | (1) |
| Womack et al., 2020 | (3) Relationships among working conditions and abuse | Survey | N = 240 (support workers) | Quantitative | Unkown | Workers | (1) |
| **Action: Intervention Studies** | | | | | | | |
| **Paper** | **Research Design** | **Data Collection Instrument** | **Sample** | **Methodology** | **Date of Data Collection** | **Abuse Towards**  **(Workers/**  **Clients)** | **Research Question Addressed *** |
| Bien et al., 2021 | Observational | Observation tool | N = 26 (home care agencies. Workers were recruited to participate from the agencies) | Qualitative | Unknown | Workers | (2) |
| Byon et al., 2017c | Observational | Questionnaire | N = 961 direct care workers in home care | Quantitative | 2006 | Worker | (2, 3) |
| Byon et al., 2020 | Observational | Survey, focus groups, interviews | N = 23 (18 home care nurses and 5 management staff) | Mixed Methods | Unknown | Workers | (2) |
| Couture et al., 2019 | Observational | Interviews and focus groups | N = 25 (social workers and clinical supervisors) | Qualitative | 2013 | Clients | (2, 3) |
| Dyer & Abildso, 2019 | Experimental | Survey | N= 125 (home care workers) | Quantitative | 2015 | Clients | (2, 3) |
| Glass et al., 2017 | Experimental | Survey | N = 306 (home care workers) | Quantitative | Unknown | Workers | (2. 3) |
| Gross et al., 2013 | Observational | Survey | N = 40 (home care branch managers) | Quantitative | 2007-09 | Workers | (2) |
| Johnson, 2015 | Observational | Semi-structured interviews | N = 16 (home healthcare nurses) | Qualitative | Unkown | Clients | (1) |
| Kim, Choi, & Yoon, 2020 | Observational | Survey and focus group interviews | N = 363 (home visiting nurses. 6 participated in focus groups. All participated in survey) | Mixed Methods | Unknown | Workers | (1) |
| MacDonald, Lang, & MacDonald, 2011 | Observational | Semi-structured interviews | N = 24 (researchers, policy makers, health care providers, and decision makers) | Qualitative | 2008-09 | Workers | (1) |
| Markkanen, Galligan, & Quinn, 2017 | Observational | Survey | N = 29 (secondary analysis of pre- and postsurveys of home healthcare providers) | Qualitative | 2004-09; 2010-14 | Workers | (1) |
| Rabold & Goergen, 2013 | Observational | Survey | N = 503 (care worker) | Quantitative | 2005 | Clients | (1) |
| Schnelli et al., 2021c | Observational | Survey | N = 852 (health care professionals) | Quantitative | 2019 | Workers | (1, 2, 3) |
| Singh, Jha, & Purbey, 2020 | Observational | Questionaire | N = 397 (nurses) | Quantitative | Unknown | Workers | (1) |
| Vladutiu et al., 2016 | Observational | Interview | N = 191 (home care and hospice care workers) | Quantitative | 2008-09 | Workers | (2) |
| *Research Question: (1) what is known about abuse experienced by both home care workers and home care clients from recent empirical research studies? And, (2) what are the current intervention strategies to prevent or reduce abuse in paid home care settings and (3) are they effective? | | | | | | | |

| **Appendix B**  *Critical Findings from Research Studies* | | | |
| --- | --- | --- | --- |
| **Paper** | **Setting**  **(Country)** | **Focus of Study** | **Key Findings** |
| Abey-Nesbit et al., 2021 | New Zealand | Identify factors associated with home care worker stress among New Zealand home care settings. | Home care workers experience stress when clients display aggressive behavioural symptoms. |
| Ayalon, 2012 | Isreal | Evaluate depressive symptoms in a sample of Filipeno home care workers. | Home care workers who experience moderate levels of abuse is asspciated with worker depression. |
| Ayalon, 2016 | Isreal | Evaluate a conceptual model for neglect among older people in home care. | The conceptual models is a reliable tool to identify neglect among older home care clients. |
| Bien et al., 2021 | United States | Describe the occupational hazards facing home health care workers. | Home care clients who display confusion were categorized as a potential risk to incite violence towards home care workers. |
| Brouillette et al., 2017 | United States | Quantify the risk of sharp objects among home care workers. | Home xare workers are at a serious risks of being injured by a sharp object during routine home care visits. |
| Byon et al., 2016 | United States | Determine the association between client history of violence and workers experiencing client violence. | Care workers who provide service to clients who have a history of abuse are likely to report situations of physical and verbal abuse. |
| Byon et al., 2017a | United States | Study the affects of language barriers on violence against home care workers. | Violence is prevalent in settings where language issues is a barrier of concern between home care workers and clients. |
| Byon, et al., 2017b | United States | Identify patterns of relationships between home care workers and patients, and assess how the relationship was associated with violence. | Situations of physical abuse is more common in scenarios where there is rapport between home care workers and clients. |
| Byon et al., 2017c | United States | Determine if patient violence is a component of job demand. | patient violence is not beleived to be a separate factor from job demand in home care. |
| Byon et al., 2020 | United States | Explore the factors of home health care nurses reporting abuse. | Home care nurses who have access to reporting procedures are more likely to report situation of abuse they experience from home care clients. |
| Capeletto et al., 2021 | Rio De Janeiro | Identify the prevalence of using restaints among older people in home care. | Physical restraints were commonly used to control home care clients who display aggressive behaviours. |
| Choi et al., 2016 | South Korea | Identify protection for home care patients in Korea by investigating accident cases. | Care workers are at an increased risk of experiencing abusive incidents from clients with senile dementia. |
| Couture et al., 2019 | Canada | Assess a screening procedure that detects mistreatment in home care settings. | Organizations should take steps to reduce the stigma of reporting abuse by prioritizing time to develop interventions aimed at reducing the prevalence of abuse in home care settings. |
| Dyer & Abildso, 2019 | United States | Evaluate the effects of a statewide intimate partner violence (IPV) training on home visitors’ perceived knowledge, skills, and abilities to address IPV experienced by their clients and examine home visitors’  perceived barriers to addressing IPV during client home visits. | Home care workers who participate in an IPV training program is responsible for a significant improvement in their knowledge, skills, and abilities to respond to situations of violence directed toward home care clients. |
| Friedman et al., 2015 | United States | Identify the prevalence of elder mistreatment of older adults receiving home care from visiting nurses. | Home care workers are not able to easily recognize the signs of neglect among clients. |
| Fujimoto et al., 2019 | Japan | Clarify nurse's experiences of violence in home settings. | Approximately half of the sample of home care workers experienced some form of abuse during their career in home care. |
| Glass et al., 2017 | United States | Examine the effectiveness of a workplace violence prevention program among female home care workers. | Care workers knowledge and confidence for responding to aggressive and abusive situations can be improved by participating in training programs. |
| Grasmo, Liaset & Redzovic, 2021 | Norway | Explore how working conditions affect the safety of home care workers. | Unpredictable working conditions put the safety of home care workers in jeapordy. |
| Green & Ayalon, 2016 | Isreal | Explore the help seeking behavours of migrant workers who experience work related violence. | Migrant home care workers are highly likely to experience workplace abuse, yet are unable to report these situations to the appropriate authorities. |
| Green & Ayolon, 2017 | Isreal | Explore what characteristics among migrant home care workers are related to abuse. | Migrant home care workers with little on-the-job experience are vulnerable to experiencing abuse. |
| Green & Ayalon, 2018 | Isreal | Assess the working conditions and the prevalence of  abuse and exploitation among live-in migrant home care workers and live-out local home care workers. | Live-in migrant home care workers and live-out local home care workers report experiencing workers’ rights violations. |
| Gross et al., 2013 | United States | Explore the content of existing programs to prevent violence in home and hospice care. | Home care workers are not required to complete training on violence prevention/intervention. |
| Hanson et al., 2015 | United States | Investigate the prevalence of violence in the workplace experienced by home care workers. | The abuse experienced by home care workers is associated with factors such as stress, depression, sleeping problems, and burnout. |
| Herron & Wrathall, 2018 | Canada | Understand how the environment incites abusive responsive behaviours among people with dementia. | Individual, interpersonal, and micro factors can be used to categorize the actions of clients living with dementia. |
| Ifediora, 2018 | Australia | Minimize the risk of distress that comes from aggression in after hour house care services. | Abuse towards doctors who perform after hours home visit services are prevalent, and affects roughly one in two doctors. |
| Johnson, 2015 | United States | Determine how nurses who work in home healthcare perceive self neglect among older people. | Nurses believe self-neglect among older people was a result of psychological reasons, such as mental illness that was undiagnosed. |
| Kim, Choi, & Yoon, 2020 | South Korea | Identify experiences of violence towards visiting nurses during home visits. | There is an insufficient organizational support system for visiting nurses experiencing violence in Korea. |
| Kim, Noh, & Muntaner, 2013 | United States | Determine emotional demands on home care workers and associate the demands with symptoms of depression. | The emotional demands experienced by home care workers include unfair treatment, abuse and emotional suppression. |
| Koivula et al., 2016 | Finland | Examine how home care clients who consume alcohol affect the work of home care workers. | Intoxicated clients make the daily work of home care workers difficult by not cooperating with their requests. |
| Kurata, & Ojima, 2014 | Japan | Assess knowledge of physical restraint techniques among home care workers and family caregivers. | Agency-hired care workers were more knowledgeable in physical restaint procedures that are prohibited and were more likely to be able to recognize harmful procedures than family caregivers. |
| MacDonald, Lang, & MacDonald, 2011 | Canada | Exlore the perspective of decision makers in issues related to safety in home care. | The four patterns identified in this study was erosion of home as a safe haven, incongruence between care demands, client vulnerabilities, and fragmentation of services. |
| Markkanen et al., 2014 | United States | Summarize occupational safety and health hazards among home care workers. | Verbal abuse towards home care workers by clients is highly prevalent and a major concern for worker’s well being. |
| Markkanen, Galligan, & Quinn, 2017 | United States | Summarize occupational safety and health risks to home infusion therapy. | The need for home infusion therapy will continue to  grow in the future. |
| Möckli et al., 2020 | Switzerland | Explore the level of work engagement and burnout among home care workers in Switzerland. | Emotional exhaustion among home care workers is directly related to job demand. |
| Nakaishi et al., 2013 | United States | Examine experiences of violence among home care workers who provide care to clients. | Barriers to report abuse, tolerance of violence, and lack of training to prevent violence increases the risk of abuse among care workers in home care settings. |
| Quinn et al., 2016 | United States | Assess occupational hazards for a wide range of homecare working conditions and compare occupational health and safety concerns with experiences of home care aides. | Home care workers frequently report experiencing abuse in home care settings. |
| Rabold & Goergen, 2013 | Germany | Determine risk factors of abuse of care recipients by nursing staff who provide home care services. | Psychological and verbal abuse is the most common forms of aggression directed towards older clients by home care workers. |
| Schilgen et al., 2019 | Germany | Identify work-related stressors among German home care nurses. | Regardless of their origin or culture, nurses perceive time pressure, lifting patients, lack of appreciation or the client's personal fate as burdening. |
| Schnelli et al., 2021a | Switzerland | Explore documented cases of aggression among home care workers in home care settings who experience cognitive impairments. | Factors associated with aggression in clients with cognitive impairment can be categorized into three themes: physical abuse, verbal abuse, and importunate bahaviour. |
| Schnelli et al., 2021c | Switzerland | Understand how organizational strucutures address aggressive incidents towards home care workers. | Organizational support promotes positive well-being among home care workers. |
| Schnelli et al., 2021b | Switzerland | Explore patterns of violence towards health professionals in home care by clients. | Verbal abuse was frequently directed towards home care workers by clients. |
| Singh, Jha, & Purbey, 2020 | India | Categorize hazards in home care that affects home care aides. | The time pressure to arrive at a clients home and rushing care practices are identified as stressors for care worker. |
| Vladutiu et al., 2016 | United States | Learn more about the workplace violence prevention and training program. | Most providers rate the quality of training as average, and efforts must be made to improve workplace violence prevention training. |
| Vu et al., 2014 | Canada | Examine the variation the characteristics of home care clients with dementia in those with neurological conditions. | Clients with dementia display agression towards home care workers, which contributes to worker distress. |
| Wang et al., 2016 | United States | Examine the characterstics of home care patients with mental health disorders. | Depression, anxiety, and abuse were common characteristics among older clients who recieve home care. |
| Womack et al., 2020 | United States | Estimate the prevalence of aggression among support workers and compare to data on home care workers. | Home care workers who are abused by their clients experience fatigue and depression. |
